# 14-3-3 σ Expression Effects G2/M Response to Oxygen and Correlates with Ovarian Cancer Metastasis

**DOI:** 10.1371/journal.pone.0015864

**Published:** 2011-01-10

**Authors:** Dashnamoorthy Ravi, Yidong Chen, Bijal Karia, Adam Brown, Ting Ting Gu, Jie Li, Mark S. Carey, Bryan T. Hennessy, Alexander J. R. Bishop

**Affiliations:** 1 Greehey Children's Cancer Research Institute, University of Texas Health Science Center, San Antonio, Texas, United States of America; 2 Department of Cellular and Structural Biology, University of Texas Health Science Center, San Antonio, Texas, United States of America; 3 Department of Epidemiology and Biostatistics, University of Texas Health Science Center, San Antonio, Texas, United States of America; Department of Gynecologic Medical Oncology, University of Texas MD Anderson Cancer Center, Houston, Texas, United States of America; 5 Division of Gynecologic Oncology, Department of Obstetrics and Gynecology, University of British Columbia, Vancouver, Canada; 6 Department of Medical Oncology, Beaumont Hospital, Dublin, Ireland; University of Medicine and Dentistry of New Jersey, United States of America

## Abstract

**Background:**

*In vitro* cell culture experiments with primary cells have reported that cell proliferation is retarded in the presence of ambient compared to physiological O_2_ levels. Cancer is primarily a disease of aberrant cell proliferation, therefore, studying cancer cells grown under ambient O_2_ may be undesirable. To understand better the impact of O_2_ on the propagation of cancer cells *in vitro*, we compared the growth potential of a panel of ovarian cancer cell lines under ambient (21%) or physiological (3%) O_2_.

**Principal Findings:**

Our observations demonstrate that similar to primary cells, many cancer cells maintain an inherent sensitivity to O_2_, but some display insensitivity to changes in O_2_ concentration. Further analysis revealed an association between defective G2/M cell cycle transition regulation and O_2_ insensitivity resultant from overexpression of 14-3-3 σ. Targeting 14-3-3 σ overexpression with RNAi restored O_2_ sensitivity in these cell lines. Additionally, we found that metastatic ovarian tumors frequently overexpress 14-3-3 σ, which in conjunction with phosphorylated RB, results in poor prognosis.

**Conclusions:**

Cancer cells show differential proliferative sensitivity to changes in O_2_ concentration. Although a direct link between O_2_ insensitivity and metastasis was not determined, this investigation showed that an O_2_ insensitive phenotype in cancer cells to correlate with metastatic tumor progression.

## Introduction

Cell lines derived from cancer patients provide an experimentally manipulable model system that facilitates investigations into cancer biology and its therapy. The unlimited proliferation potential of cancer cells is a major hallmark of malignancy, however the use of standard tissue culture protocols often restricts cell proliferation, as observed with primary cell lines [1], [2], [3], [4]. Although the use of physiological conditions is known to impact *in vitro* proliferation of cancer cells [5], [6], [7] and primary cells are known to propagate better at physiological O_2_, the impact of physiological O_2_ on *in vitro* cancer cell proliferation is relatively unexplored. However, it has been reported that altered concentrations of O_2_ results in clear differences in cell proliferation and response to drug treatment in the cancer cells [8], [9], [10].

Oxygen, in addition to nutrients and growth factors, is vital for proper cell growth and its availability has a direct impact on cellular metabolism, signaling pathways, proliferation, differentiation and survival [3], [11], [12], [13]. Many *in vitro* investigations have demonstrated the advantages of physiological O_2_ for tissue culture. For example, the biological behavior of primary cell cultures with a physiological concentration of O_2_ (2.7–5.3%) is far superior compared to the standard practice of growing cells under atmospheric or “ambient” O_2_ concentration (21% O_2_) [4]. In fact, these two growth conditions are known to result in distinct metabolic and molecular characteristics [13].

The importance of considering O_2_ tension in cancer biology is well established. For example, the fact that many cancers exist in a ‘hypoxic’ state has led to the development of hypoxia-targeted therapy [14], [15]. In general the hypoxic concentration of O_2_ is <1% for most solid tumors, however the hypoxic concentration could vary based on the cell types and the normal perfusion status [16] and additionally, hypoxia tends to inhibit cell proliferation [17]. Physiological O_2_ tension varies from 2.7–5.3% in the interstitial space [18], where many primary tumors reside, to 14.7% in the arterial circulation and lungs, where migrating and potentially metastatic cancer cells are often found. Therefore, cancer studies that are only conducted in ambient (21%) O_2_ may miss pertinent biological observations. This may be particularly important when attempting to study the progression of cancer to metastatic disease, which is a significant event in cancer etiology and is associated with poor prognosis [19]. Considering the differences in O_2_ tension in different compartments of the body, an understanding of the effect of O_2_ concentration on cancer cell proliferation could provide useful insights into the mechanisms involved in the pathological progression of cancer.

Cancer cells that have acquired mutations in either oncogenes or tumor suppressor genes display a characteristic uncontrolled proliferation phenotype [20]. For example, tumor suppressors such as p53 or RB act as “molecular gatekeepers” known to affect cell cycle progression. Mutation of such factors facilitates unlimited proliferation in cancer cells [20]. Cell cycle progression involves a sequential series of events catalyzed by cyclins and cyclin-dependent kinases (CDKs) [21], and in normal cells is a tightly regulated process. The tumor suppressor p53 is a master regulator of G1/S and G2/M phase transition in the cell cycle [22] and is known to have an important role in responding to oxygen concentration, particularly hypoxia (<1% O_2_) [23] or hyperoxia (95% O_2_) [24]. Although examining the effect of extreme O_2_ conditions is both important and revealing, it must be noted that these previous studies did not investigate the response of p53 at physiological (3%) O_2_ and ambient (21%) O_2_. p21 and 14-3-3 σ are transcriptional targets of p53 that are involved in regulating G1/S and G2/M transitions of the cell cycle by targeting CDK2 and CDC2 (also known as CDK1), respectively [22], [25]. The CDKs, in turn, regulate RB protein function, to mediate cell cycle progression through G1/S and G2/M [26]. Therefore, disruption of RB function could also impact the control of cell cycle progression [26]. Considering that differences in O_2_ concentration result in altered cell cycle progression in primary cells but cancer cells frequently display cell cycle control defects, there is clearly the potential that these defects may impact how cancer cells respond to altered O_2_ levels in a manner that could have a profound influence on cancer progression.

Here we examined the biological behavior of ovarian cancer cells under physiological and ambient O_2_. Interestingly, some of the ovarian cancer cell lines had a normal response to O_2_ concentration, (*i.e.* reduced cell proliferation with increased O_2_ concentration) while the proliferation of other ovarian cancer cell lines was unaffected by this O_2_ increase. Further, our investigations revealed that 14-3-3 σ and its role in the cell cycle influence the proliferative response to altered O_2_ levels. Considering the variation in partial pressure of oxygen throughout the body and the potential importance that this context may have on cancer progression, it is crucial to understand the affect of O_2_ concentration on cancer cell proliferation and cancer progression. We provide evidence that acquisition of O_2_ insensitivity may be a component in cancer progression and a hallmark of successful metastatic disease.

## Results

### Physiological oxygen results in increased cell proliferation in ovarian cancer cells

**Figure 1:**
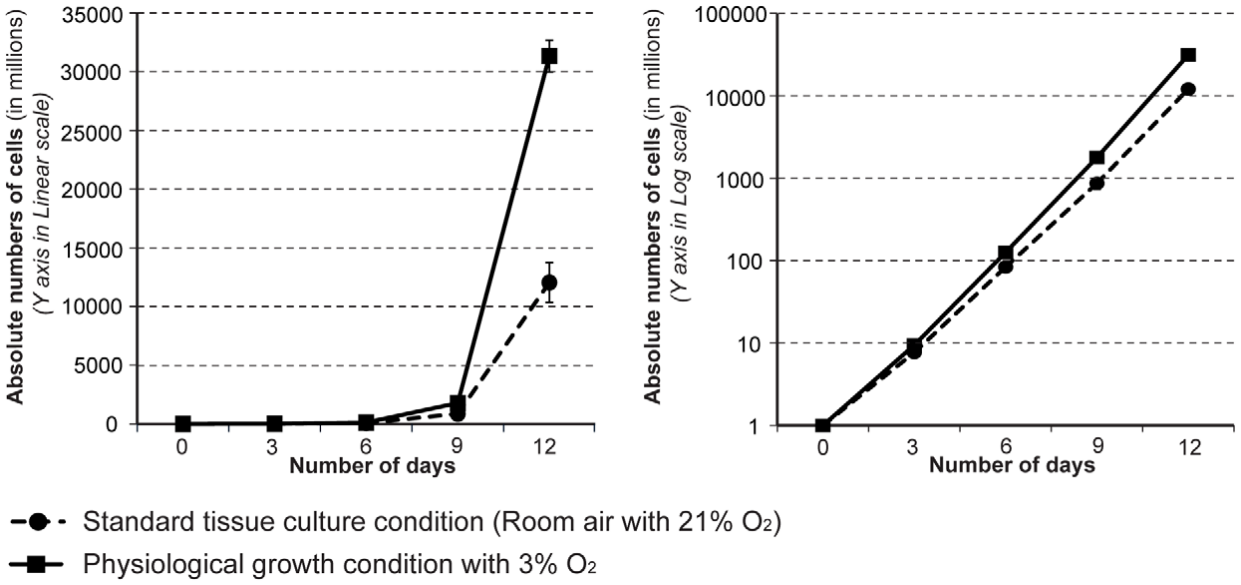
Cancer cell proliferation is markedly suppressed by the standard cell culture conditions used for *in vitro* experiments. Equal numbers of A2780 ovarian cancer cells were seeded in a 10 cm petri dish and were routinely maintained under 3% O_2_ (physiological) or 21% O_2_ (ambient). The increase in cell numbers was determined by counting manually once in three days, and the total cell numbers were estimated and plotted using linear scale (in Graph A) and log scale (in Graph B).

**Figure 2:**
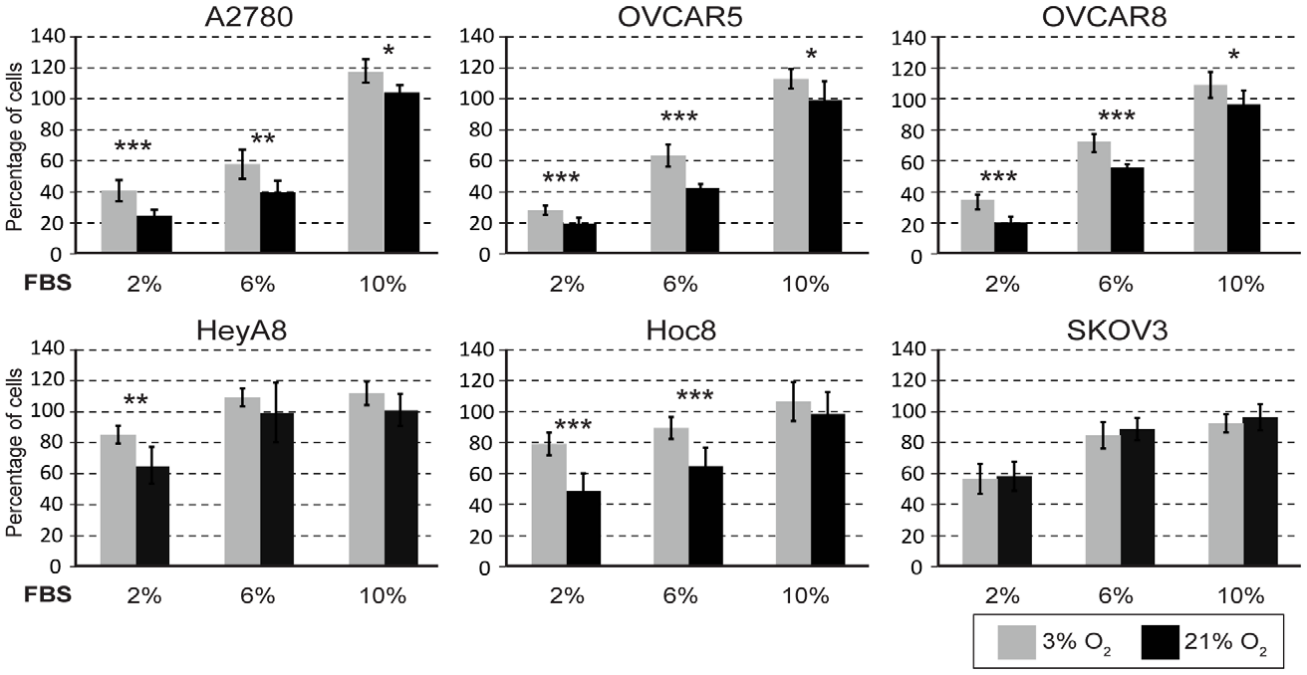
Ovarian cancer cells grown under physiological and ambient O_2_ show differential proliferation response. Ovarian cancer cell lines were cultured under 3% or 21% O_2_ and the extent of proliferation was determined following 3 days of growth (see Materials and Methods section). For each cell line, the percent of cell proliferation at 3% O_2_ (light shaded bars) and at different concentrations of serum was compared with proliferation under standard tissue culture conditions consisting of 21% (ambient) O_2_ (dark shaded bars) and 10% FBS. The error bars represent the standard deviations of mean and statistical significant (by student T Test) differences in proliferation between 3% and 21% O_2_ for each concentration of serum is indicated by an asterisk [(*) *p*<0.05, (**) *p*<0.001 and (***) *p*<0.0001].

In our initial studies we compared the effect of physiological (3% O_2_) and ambient (21% O_2_) oxygen concentration using A2780 ovarian cancer cells and observed that 12 days of cell culture under these conditions resulted in a 2.6 fold growth suppression under 21% O_2_ (Figure 1). Therefore, we examined the affect of O_2_ concentration on the growth potential of six ovarian cancer cell lines using physiological (3% O_2_) and ambient (21% O_2_) oxygen concentrations. Since the serum present in cell culture medium can also have a dominant influence on growth, we also tested the affect of various concentrations of serum. Regardless of the amount of serum present in the growth medium, culturing in 21% O_2_ generally resulted in a significant decrease in cell proliferation for four of the ovarian cancer cell lines (A2780, OVCAR5, OVCAR8 and HOC8) compared to 3% O_2_ (Figure 2). The only exception observed was with HOC8 cells in the presence of the highest concentration of serum (10% v/v), where an insignificant O_2_-dependent growth effect was observed (Figure 2). Presumably the lack of response in Hoc8 results from a dominant influence of serum, which was not observed with A2780, OVCAR5 and OVCAR8. In contrast, there was no significant effect on the growth of SKOV3 and HeyA8 cell lines by increasing the O_2_ concentration to 21%, irrespective of serum concentrations (Figure 2). The observed exception was HeyA8 cultured under 2% serum, which showed decreased cell proliferation at 21% O_2_ compared to 3% O_2_ (*p*<0.001). In contrast to the effect of O_2_ levels, increasing the concentration of serum resulted in a proportional growth increase in the ovarian cancer cell lines A2780, OVCAR5 and OVCAR8 (*p*<10^−5^, Figure 2). The concentration of serum had a moderate influence on growth in SKOV3 and HeyA8 (Figure 2); a serum concentration between 2 and 6% had a significant effect (*p*<10^−5^) in SKOV3, while HeyA8 serum concentration between 2 and 10% serum had the greatest effect at 3% O_2_ (*p*<10^−5^) (Figure 2). Increasing serum concentration from 6% to 10% had little effect on growth of HeyA8, SKOV3 and HOC8 (Figure 2). Together, it appears that both oxygen levels and serum concentration affect the growth of these ovarian cancer cell lines, but in an independent fashion. As expected from work by others with primary cells [4], we observed that the majority of the ovarian cancer cells displayed decreased cell proliferation at ambient O_2_ concentration compared to physiological O_2_ concentration. However, two cell lines did not appear to have inhibited cell proliferation at the higher (ambient) O_2_ levels. We therefore categorized the ovarian cancer cell lines based on these differences, being either O_2_ sensitive (A2780, OVCAR5, OVCAR8 and HOC8) or insensitive (SKOV3 and HeyA8) (Figure 2). Overall, these differences suggest heterogeneity in growth regulation responses to physiological cues of O_2_ levels in these cultured cell lines.

It is possible that the apparent O_2_ insensitivity and differences in proliferation resulted from differences in the doubling time of each cell line. For example, if SKOV3 and HeyA8 (the O_2_ insensitive cell lines) proliferate more slowly, O_2_ dependent proliferation changes may be too trivial to measure. Therefore, we measured the cell doubling time for all ovarian cancer cell lines. Our results showed that under standard tissue culture conditions (10% serum and 21% O_2_) the doubling time for all ovarian cancer cell lines were somewhat similar (<24 hours) except for HOC8, which had an extended doubling time of about 45.5±4.9 hours (Table S1, and see Methods S1). Therefore, most of the ovarian cancer cell lines were dividing at an approximately equal rate, and gross difference in doubling time is unlikely to be a factor in the observed proliferation differences between cell lines under different conditions.

### Oxygen sensitivity correlates with dynamic changes in the S and G2 phases of the cell cycle

**Figure 3:**
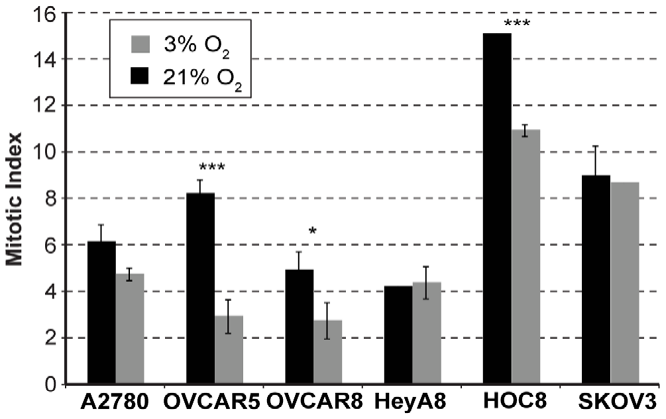
Mitotic index in the ovarian cancer cell lines grown under 3% or 21% O_2_. Mitotic index in the ovarian cancer cell lines that were cultured under 3% or 21% O_2_ for 3 days were determined by counting nuclei with condensed chromosomes, among the minimum of 1000 cells present in each experiment. Statistical significance was determined by ANOVA and the significant difference in the mitotic index between 3% and 21% O_2_ is denoted by an asterisk [(*) *p*<0.05, (***) *p*<0.0001].

Considering the differences in proliferation observed for ovarian cancer cell lines grown under either 3% or 21% O_2_, we examined whether O_2_ concentration alters the cell cycle profile of each cell line. Irrespective of serum concentration, comparing 3% O_2_ to 21% O_2_ resulted in a significant decrease in the percentage of cells that were in the G1 phase of the cell cycle and a significant increase in the percentage of cells in S phase (Table 1), which was expected based on previous observations made with primary cells [27]. Furthermore, in three of the O_2_ sensitive cell lines (A2780, OVCAR5 and OVCAR8) the percentage of the cell population in the G2 phase was increased significantly in 21% O_2_. However a significant increase in G2 was not observed in the fourth O_2_ sensitive cell line, HOC8 (Table 1). Similar to HOC8, the O_2_ insensitive cell lines, SKOV3 and HeyA8, did not display a significant alteration in the proportion of cells in the G2 phase of the cell cycle when grown under 3% O_2_ or 21% O_2_ (Table 1). Considering that the O_2_ sensitive cell lines proliferated more slowly at 21% O_2_ compared to 3% O_2_ despite having smaller proportions of their cell population in G1 and a increased proportions in S and G2, we conclude that these cells must be progressing more slowly through the cell cycle. However, for the O_2_ insensitive cell lines and HOC8 (with the significantly extended doubling time), we did not observe a significant increase in the percentage of cells in G2 when the O_2_ levels were increased. These results suggest that although the G1 and S phases of the cell cycle are responding similarly to changes in O_2_ concentration in both O_2_ sensitive and insensitive cell lines, it is the G2 phase of the cell cycle that is not responsive to O_2_ concentration in the O_2_ insensitive cell lines. Therefore, the difference in cell cycle response observed with these ovarian cancer cell lines might be at the level of regulation during the cell cycle progression from G2 to M phase. It is also possible that the changes observed with G2 and O_2_ sensitivity in these cancer cell lines is reflected in the mitotic component of the cell cycle. Our observation of the mitotic cells present in the O_2_ sensitive and insensitive cell lines grown under 3% and 21% O_2_ supports this conclusion; the O_2_ sensitive cell lines show a proportionate decrease in the mitotic cell population observed at 21% O_2_ compared to 3% O_2,_ (Figure 3), corresponding to an accumulation of cells at G2 at 21% O_2_ (Table 1). Similarly, in the O_2_ insensitive cell lines (HeyA8 and SKOV3) the proportion of mitotic cells remained unaltered regardless of O_2_ concentrations (Figure 3). This is expected because, as noted previously (Table 1), the proportion of cells at G2 in the O_2_ insensitive cell lines were also unaffected by O_2_ concentration. We conclude that most cancer cells retain an ability to regulate cell cycle in response to changes in O_2_ concentration comparable to wild type cells [27]. However, some cancer cells may lose O_2_ concentration dependent control of cell cycle (as in the O_2_ insensitive cancer cell lines), resulting in a distinct phenotype.

**Table 1:** FACS profile for cell cycle analysis with ovarian cancer cells that were grown under cell culture conditions consisting of increasing serum and O_2_ concentration.

Cells	Serum	G1				S			G2		
		3% O_2_	21% O_2_		*p*-value	3% O_2_	21% O_2_	*p*-value	3% O_2_	21% O_2_	*p*-value
	2%	66.8±0.9	62.5±0.2		0.0011	25.6±1	27.5±0.1	0.02800	7.6±0.4	9.96±0.1	0.0007
A2780	6%	76.9±2.1	64.3±0.7		0.0006	17.5±0.9	29±0.1	<0.0001	5.6±1.4	6.7±0.6	** *NS* **
	10%	84.1±3.2	69.5±1.4		0.0028	14.3±1.9	26.5±1.3	0.0080	1.5±1.3	4±0.7	0.0411
	2%	66.4±0.7		60.2±0.4	0.0001	25.8±0.2	29.3±0.6	0.0006	7.7±0.5	10.4±0.3	0.0017
OVCAR5	6%	79±1.5		70.2±0.5	0.0014	17.4±1.5	25.1±0.9	0.0015	3.4±0.4	4.7±0.6	** *NS* **
	10%	90.6±1.2		84±1.6	0.0077	8.2±1.4	14.6±1.5	0.0052	1.1±0.1	1.2±2.1	** *NS* **
	2%	74.7±10.7	69±1		** *NS* **	15.5±2.5	23.7±0.6	0.0052	13.7±2.3	6±0	0.0043
OVCAR8	6%	66±1	59±1		0.0010	25.7±1.5	30±0	0.0079	7±0	9.7±0.6	0.0013
	10%	69±4.6	61.7±1.5		0.0582	24.3±1.5	30.7±1.2	0.0045	5.3±3.8	6.3±0.6	** *NS* **
	2%	69.3±1.5	58.7±2.1		0.0020	25±1	34±1	0.0003	4.3±1.5	5.7±1.5	** *NS* **
HeyA8	6%	62.7±0.6	53.7±1.5		0.0006	30.3±1.2	37.3±2.5	0.0118	5.3±0.6	8±3.5	** *NS* **
	10%	61.7±1.2	50.3±2.3		0.0016	30.7±1.2	37.3±1.2	0.0021	6.7±2.3	11±1.6	** *NS* **
	2%	80±1.7	72.3±1.5		0.0045	15±0	18.7±1.5	0.0141	5±1.7	8±1	** *NS* **
HOC8	6%	80.3±1.2	72.7±1.2		0.0012	15.3±1.5	22.7±1.5	0.0041	3.3±1.5	3.7±1.5	** *NS* **
	10%	78.7±2.5	73.3±0.6		0.0232	16.3±0.6	23.7±0.6	<0.0001	4.3±2.5	3±1	** *NS* **
	2%	74±2	62.1±1.7		0.0014	13.5±3.5	27.7±1.2	0.0026	12±2	10.3±2.5	** *NS* **
SKOV3	6%	76.7±1.5	65.3±3.2		0.0052	14±1	25.7±1.5	0.0003	9.3±1.5	9±2	** *NS* **
	10%	80.3±1.2	68±1.7		0.0005	12.7±1.5	26±1	0.0002	7±1	6.3±0.6	** *NS* **

**NS** “Not Significant”

### Oxygen insensitivity correlates with altered G2/M components

**Figure 4:**
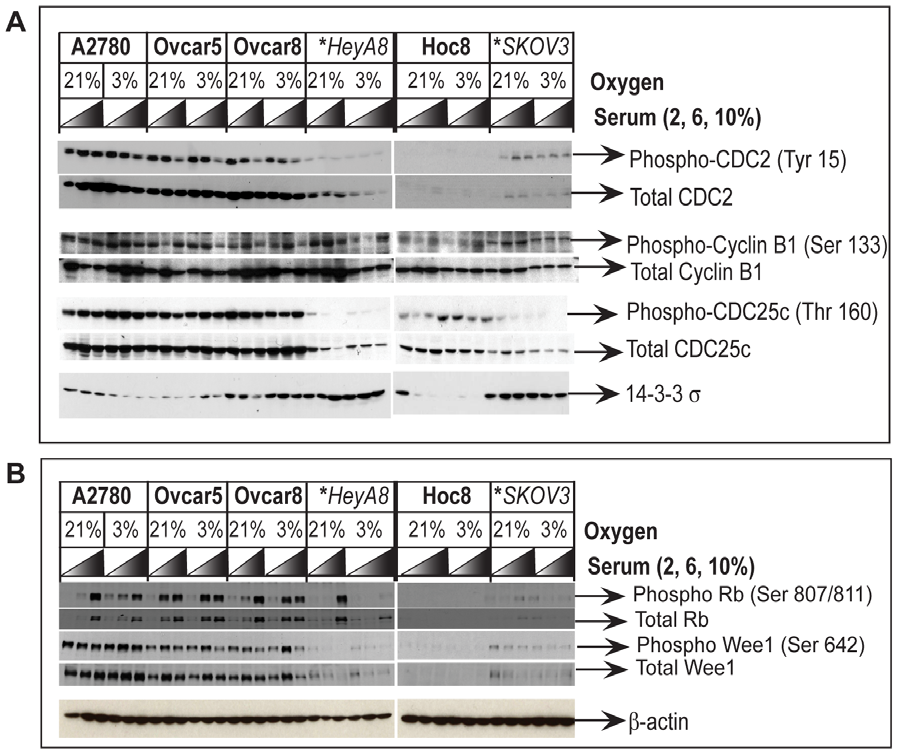
Western blot analysis of G2 cell cycle regulatory proteins and the relevance to O_2_ sensitivity in the ovarian cancer cell lines. Protein lysates prepared from the ovarian cancer cell lines maintained in growth medium consisting of increasing concentrations of serum and 21% or 3% O_2_ were analyzed by Western blot. (A) Compared to O_2_ sensitive cell lines, decreased expression of the core components involved in G2/M cell cycle progression CDC2/cyclin B1 complex and its activator CDC25c is observed in the O_2_ insensitive cell lines (indicated by asterisk and italics), while the expression of 14-3-3 σ, a protein that inhibits CDC2 is elevated in the O_2_ insensitive cell lines. (B) Phosphorylation of RB and Wee1 were monitored as an indicator for CDC2 function because both RB and Wee1 are known targets for phosphorylation by CDC2. Equal loading of protein extracts were monitored by probing the stripped Western blots with the primary antibody for β-actin.

Thus far we have demonstrated that O_2_ sensitive cell cycle response at the G2/M transition is lacking in the O_2_ insensitive cell lines. We therefore went on to characterize this observation further by determining what component of G2/M regulation is deficient in the O_2_-insensitive cancer cells. The major effector of G2/M transition is CDC2 [22], [28]. CDC2 forms a complex with cyclin B [29], [30], which phosphorylates various structural proteins resulting in the collapse of the nuclear envelope, condensation and segregation of chromosomes [30], [31] and inactivation of other cell cycle regulatory proteins such as WEE1, RB and CDC25C [30], [32]. In normal cells, the overall levels of CDC2 protein are kept constant throughout the cell cycle [33] and are regulated by post-translational modification [33] and cellular localization [30], [31]. Once the Tyr15 residue on CDC2 is dephosphorylated by CDC25C, activated CDC2 forms a complex with cyclin B, accumulates in the nucleus, and promotes the G2/M transition[30], [33], [34]. This occurs in a stepwise fashion through increasing amounts of nuclear CDC2 protein [30]. Our examination of total CDC2 protein and phosphorylated CDC2 protein revealed that both are considerably lower in the O_2_-insensitive cell lines (HeyA8 and SKOV3) compared to the O_2_-sensitive cell lines (Figure 4A). Although the levels of CDC2 were relatively high in the O_2_-sensitive cell lines (A2780, OVCAR5 and OVCAR8) (Figure 2), we observed a decrease in Tyr15 phosphorylation status regardless of O_2_ concentration for A2780, OVCAR5 and OVCAR8 with increasing serum levels (Figure 4A). This correlates with the observation that increasing serum concentration causes increased cellular proliferation and results in a concomitant reduction in the proportion of cells in G2/M (compare with Table 1). However, no overt O_2_-dependent alteration in either total or phosphorylated cyclin B or CDC25C was observed in the O_2_ sensitive cell lines (A2780, OVCAR5, OVCAR8 and HOC8) compared to O_2_ insensitive cell lines (HeyA8 and SKOV3) (Figure 4A). Therefore, it appears that the observed decrease in the cell population in G2 in 21% O_2_ might not be dependent on phosphorylation-mediated inactivation of CDC2. It should be noted that these experiments were performed in asynchronously growing cells, and therefore it is possible that transient differences in CDC2 status were missed. Interestingly, the levels of CDC2, Cyclin B and CDC25c (the negative regulator of CDC2) were considerably lower in O_2_ insensitive cell lines (HeyA8 and SKOV3) compared to O_2_ sensitive cell lines (A2780, OVCAR5, OVCAR8 and HOC8) (Figure 4A). These observations suggest an inherent deficiency in the core components involved in the G2/M progression in the O_2_ insensitive cell lines.

p53, p21 and 14-3-3 σ are factors which have the ability negatively to influence CDC2 activity and G2/M transition [22]. Current understanding is that p53 and p21 influence cell cycle in hypoxic and hyperoxic conditions [23], [24], [35], [36]. Considering the reduced levels of CDC2 and the apparently defective G2/M checkpoint in the O_2_ insensitive cell lines (HeyA8 and SKOV3), we explored the possibility that impairment was due to a defect in any of these molecular regulators. Western blot analysis found p53 and p21 to be overexpressed in one O_2_-insensitive cell line (HeyA8). However, both were absent in the other O_2_-insensitive cell line (SKOV3), and the expression pattern for these proteins remained unaltered regardless of changes in O_2_ or serum concentration (Figure S1), suggesting that neither p53 nor p21 is relevant to CDC2’s function in O_2_ sensitivity. Interestingly, we observed a considerable elevation in the expression of 14-3-3 σ (Figure 4A) in the O_2_ insensitive cell lines (HeyA8 and SKOV3) compared to the O_2_-sensitive cell lines. Although, the level of 14-3-3 σ expression was considerably lower in all O_2_-sensitive cell lines compared to HeyA8 and SKOV3, we did observe an increase in the expression of 14-3-3 σ at 21% O_2_ with A2780 (Figure 4A). Although contradictory to the known inhibitory role of 14-3-3 σ on CDC2 activity, we concluded that high levels of 14-3-3 σ combined with reduced levels of CDC2 in a proliferating cancer cell may indicate a lack of control of G2/M progression in response to O_2_ levels.

To clarify the consequence of the low levels of CDC2 protein observed in the O_2_-insensitive cell lines, we determined the functional activity of the remaining CDC2 by examining the phosphorylation of two of its substrates, RB and WEE1. Phosphorylation of RB at the Ser 807 residue is mediated by CDC2 [32], and we observed this phosphorylation regardless of CDC2 levels or O_2_ levels with 10% serum for all cell lines except HOC8 (Figure 4B), indicating unimpaired CDC2 activity in these cell lines. A reduction in phosphorylated RB correlated with reduction of serum concentration (Figure 4B) and correlated with increased accumulation of total RB in the O_2_-sensitive cell lines (A2780, OVCAR5 and OVCAR8), but not in HOC8 (Figure 4B). Total RB was barely detectable in the O_2_-insensitive cell lines (HeyA8 and SKOV3) (Figure 4B), with the exception of 2% serum at 3% O_2_ condition in the HeyA8 cell line. Interestingly, a comparison between the RB expression pattern (Figure 4B) and cell proliferation (Figure 2) revealed that HOC8, HeyA8 and SKOV3 cells grow better in cell culture medium with a low concentration of serum (2%) compared to A2780, OVCAR5 and OVCAR8. It therefore appears that the total RB protein level response remains intact in O_2_-sensitive cell lines and that this response is probably more relevant to serum concentrations than O_2_ levels. The other target for CDC2-mediated inactivation by phosphorylation is WEE1, which can also reciprocally inhibit CDC2 function by phosphorylation [37]. We observed increased phosphorylation of WEE1 in the O_2_ sensitive cell lines (A2780, OVCAR5 and OVCAR8), barely detectable levels in HOC8, (Figure 4B) and a complete absence in the O_2_-insensitive cell lines (HeyA8 and SKOV3, Figure 4B). This pattern was largely recapitulated for total WEE1 protein levels (Figure 4B). Therefore, the absence of phospho-WEE1 in the O_2_-insensitive cell lines does not indicate an absence of CDC2 activity, but rather an absence of the WEE1 substrate. From these results we concluded that despite the reduced amounts of CDC2 in the O_2_-insensitive cell lines, CDC2 is functionally active and uninhibited by the increased levels of 14-3-3 σ. It should be noted that RB and CDC2 act upon each other to regulate each others function [38], and phosphorylation status of RB [26] or CDC2 [39] could influence E2F mediated expression of cyclins that are essential for cell cycle progression. Therefore, considering this complex relationship between RB and CDC2, the phosphorylation pattern of RB is insufficient to predict G2/M progression.

In summary, the O_2_-sensitive cell lines (A2780, OVCAR5 and HOC8) showed increased expression of CDC2 and cyclin B combined with low level of 14-3-3 σ expression. This suggests that the cell cycle components required for a dynamic proliferative response to differences in the O_2_ concentration is present in these cell lines. However in the O_2_-insensitive cell lines that express high levels of 14-3-3 σ and low levels of CDC2 and CDC25C such a dynamic cell cycle response to changes in O_2_ concentration could be impaired. We therefore pursued the possibility that this inverse correlation between 14-3-3 σ and CDC2 might be important for the O_2_-sensitive regulation of G2/M transition.

### 14-3-3 σ and mitotic progression in oxygen sensitivity

**Figure 5:**
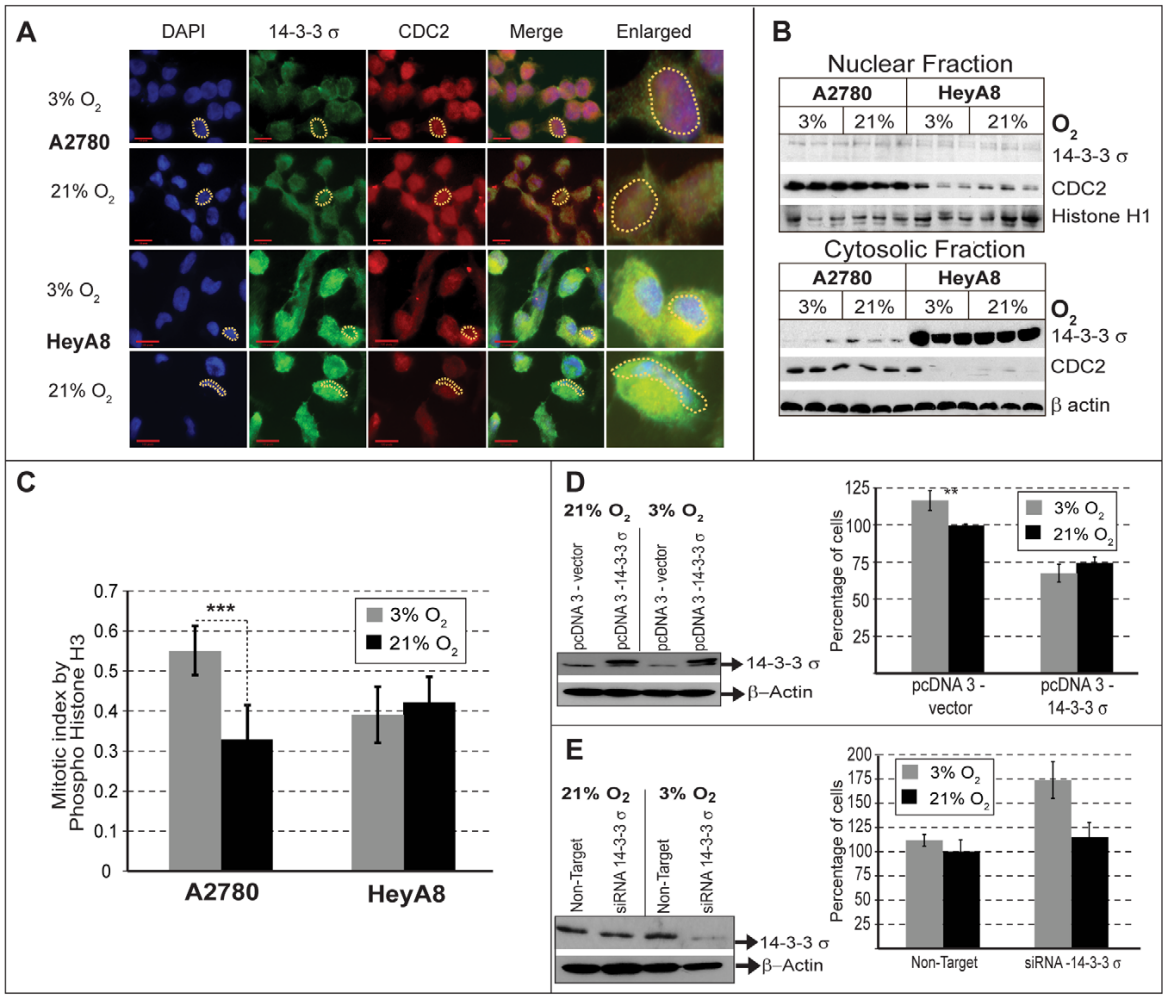
14-3-3 σand O_2_ sensitivity. (A) Cellular localization by immunofluoresence shows that 14-3-3 σ (Green) is located in the cytoplasm and CDC2 (Red) is present in the nucleus (Blue). Compared to O_2_ sensitive A2780 cells, the level of 14-3-3 σ is higher and CDC2 is low in the O_2_ insensitive HeyA8 cells. In the O_2_ sensitive A2780, 14-3-3 σ is localized both in the nucleus and cytoplasm at 21% O_2_. (A dotted yellow line, outlines a representative nuclei to indicate relative localization of 14-3-3 σ and CDC2 in these cells). (B) Western blot analysis of nuclear and cytoplasmic fractions show low levels of 14-3-3 σ in the nucleus compared to cytoplasm, with increased amounts of 14-3-3 σ being present in the cytoplasm of the O_2_ insensitive HeyA8 cells. The level of CDC2 is higher both in the nucleus and cytoplasm of the O_2_ sensitive A2780, but present in lower amount only in the nucleus of O_2_ insensitive HeyA8 cells. Histone H1 and β−actin were used as loading controls for nuclear and cytoplasmic fractions, respectively. (C) Mitotic cells were determined by counting the cells that stained positively for a mitosis specific marker, Phospho-Histone H3 from the total cell population. Mitotic fractions present at 3% or 21% O_2_ were counted in both A2780 and HeyA8 and represented as bar graph. A significant increase in mitotic index (p>0.001, indicated by asterisk) was observed in the O_2_ sensitive A2780 at 3% O_2_, but not in the O_2_ insensitive HeyA8 cells. (D) Over-expression of 14-3-3 σ in the O_2_ sensitive A2780 (Western Blot) results in loss of O_2_ sensitivity (Bar graph). For the cells transfected with empty vector (mock transfection) or 14-3-3 σ over-expression construct, the percent of cell proliferation was compared with proliferation of mock transfected cells grown under standard tissue culture conditions consisting of 21% O_2_ (ambient), and (E) in the converse experiment performed with O_2_ insensitive HeyA8, reducing the levels of 14-3-3 σ by siRNA (Western blot) results in restoration of O_2_ sensitivity (Bar graph). For the cells transfected with scrambled siRNA (mock transfection) or siRNA against 14-3-3 σ, the percent of cell proliferation was compared with proliferation of mock transfected cells grown under standard tissue culture conditions consisting of 21% O_2._

Our previous observations suggest an association between elevated level of 14-3-3 σ and O_2_-insensitivity that needs to be confirmed. Therefore, we wanted to confirm that 14-3-3 σ does indeed affect O_2_-dependent proliferation. For this part of the study, we restricted our analysis to two cell lines with wild type p53: the O_2_-sensitive A2780 [40], and O_2_-insensitive HeyA8 cell lines [41]. So far we have used Western blot analysis to monitor the overall expression levels of 14-3-3 σ and CDC2 (Figure 4A). However, since the functional responses of these proteins are dependent on their cellular localization, we used immunofluorescence to determine their cellular location under 3% O_2_ and 21% O_2_. In the O_2_-sensitive A2780 cell line, the localization of 14-3-3 σ was restricted to the cytoplasm under 3% O_2_ (Figure 5A), but was found in both the nucleus and cytoplasm at 21% O_2_ (Figure 5A). CDC2 was distributed throughout the cell and its localization was unaffected by O_2_ concentration. It therefore appears that nuclear exclusion of 14-3-3 σ correlates with a decreased fraction of cells in the G2/M phase and an uninhibited cell cycle progression when A2780 is grown at 3% O_2_, as noted before (Table 1). In contrast, the O_2_-insensitive HeyA8 cell line showed high levels of 14-3-3 σ and low levels of CDC2 (Figure 4A), with a considerable amount of 14-3-3 σ in the cytoplasm (Figure 5A). Further, 14-3-3 σ remained excluded from the nucleus even at 21% O_2_ in the HeyA8 cells (Figure 5A). These observations were further verified by Western blot analysis of nuclear and cytosolic cell fractions obtained from these cells (Figure 5B). Finally, to confirm the effect on G2/M transition, we determined the proportion of those cells in M phase for different O_2_ concentrations using the mitosis specific marker phospho-histone H3. In the O_2_-sensitive A2780 cells, under 21% O_2_, we observed a decrease in the mitotic index (P<0.001), compared to 3% O_2_ (Figure 5C). No such O_2_-dependent change in mitotic index was observed for the O_2_-insensitive HeyA8 cells (Figure 5C). These results support our initial conclusion, that the O_2_-insensitive cells lines have a deficiency in regulating cell cycle progression at G2/M in response to increased O_2_ levels (Figure 2).

The levels and cellular localization of 14-3-3 σ correlate with O_2_-sensitive proliferation. To demonstrate a direct relationship, we examined whether over-expression of 14-3-3 σ could render O_2_-sensitive A2780 cells insensitive to O_2_ and conversely whether reducing the levels of 14-3-3 σ in O_2_-insensitive HeyA8 cells could restore O_2_-sensitivity. Transient over-expression of 14-3-3 σ in A2780 cells reduced cell proliferation (Figure 5D) and resulted in loss of O_2_-sensitivity. Therefore, merely increasing 14-3-3 σ expression results in its inability to regulate G2/M in the absence of any further genetic alterations. Conversely, RNAi-mediated silencing of 14-3-3 σ expression in HeyA8 cells (Figure 5E - Western blot) resulted in a substantial increase in proliferation under 3% O_2_ (Figure 5E - Bar graph). Interestingly, when the cells from the same siRNA transfection were placed at 21% oxygen, 14-3-3 σ protein expression was induced, reducing the knockdown effect of the siRNA. This observation also suggests an O_2_-dependent transcriptional response by 14-3-3 σ. Despite this transcriptional response, we still observed a muted growth phenotype at 21% O_2_ under these conditions. Together these experiments demonstrate that 14-3-3 σ is a critical factor for controlling ovarian cancer cell proliferation in response to O_2_ concentration.

### 14-3-3 σ is frequently highly expressed in ovarian cancer and its ineffectiveness in controlling CDC2 is relevant to ovarian tumor pathology

**Figure 6:**
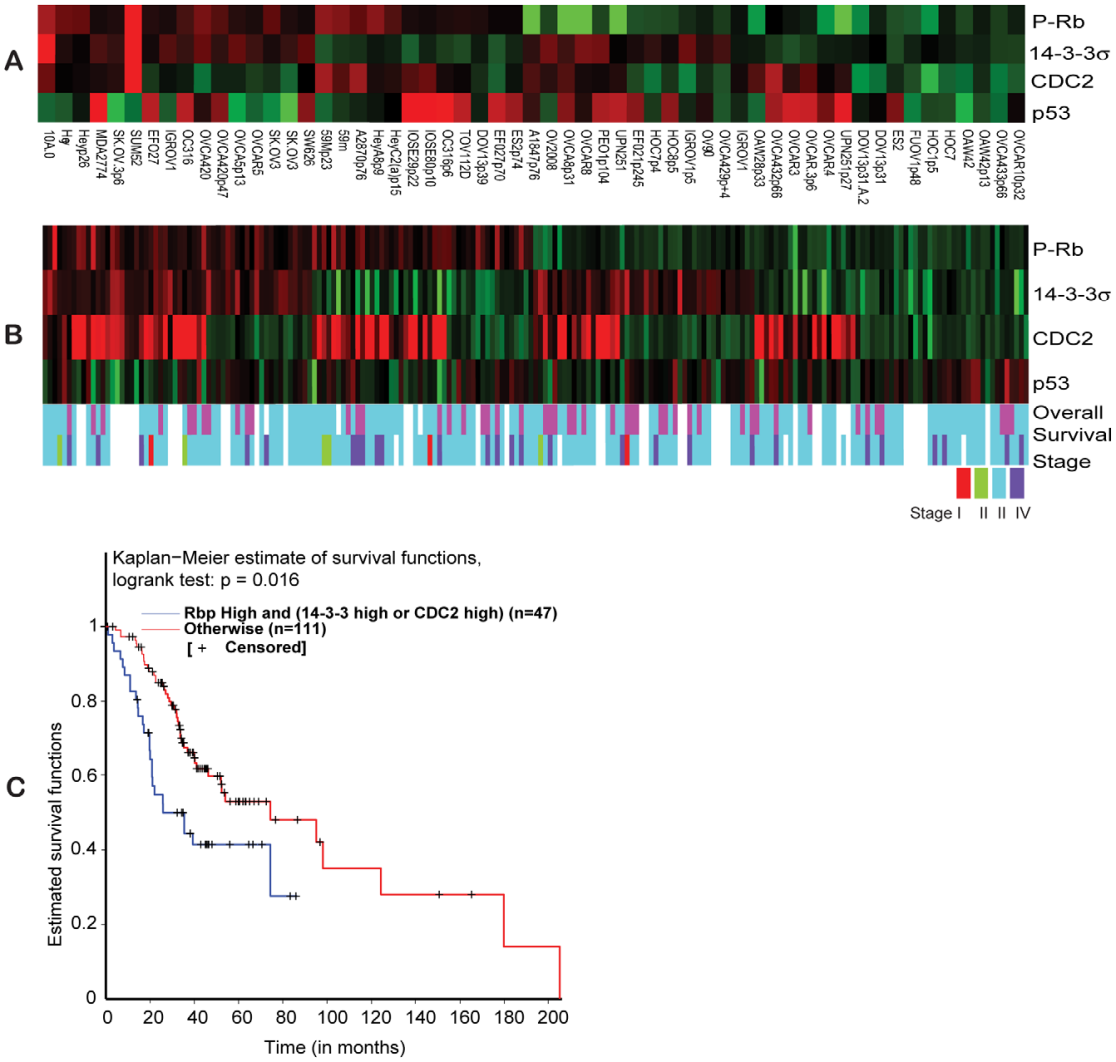
Reverse phase protein array data analysis. (A) Hierarchical clustering of normalized RPPA data over Phospho-RB (Ser 807/811), 14-3-3 σ, CDC2 and p53 across 57 ovarian cancer cell lines. (B) Hierarchical clustering of normalized RPPA data over Phospho-RB (Ser 807/811), 14-3-3 σ, CDC2 and p53 across 205 ovarian tumors. The color codes for overall survival represents overall survival >24 months (blue) and overall survival <24 months (pink). The color codes for tumor stage represent stage I (red), stage II (green), stage III (light-blue) and stage IV (dark-blue). (C). Kaplan-Meier survival curve for the RPPA results comparing the group of ovarian tumors with high Phospho-RB and high 14-3-3 σ or CDC2 (blue line) with other expression profiles (red line).

Considering that increased expression of 14-3-3 σ provides some indication of impaired G2/M control, it is possible that cancer cell lines that express high levels of 14-3-3 σ are O_2_-insensitive. The O_2_-insensitive ovarian cancer cell lines we have thus far characterized have high 14-3-3 σ (Figure 5A) and low CDC2 protein levels. It is conceivable that the same phenotypic defect might result from cells with unchecked CDC2 activity, irrespective of 14-3-3 σ levels. To determine the frequency of commonly available cancer cell lines that have the hallmarks of O_2_-insensitivity, we used a reverse phase protein array (RPPA) and screened 57 different ovarian cancer cell lines for the levels of 14-3-3 σ and CDC2, as well as phospho-RB as an indicator of CDC2 activity. Cell lines with the same name but from different labs or different passages were considered to be different. We therefore set the analysis criteria on the RPPA array to detect high phospho-RB (P-RB) and either high 14-3-3 σ or high CDC2. In the context of high levels of P-RB, this criteria should indicate that either 14-3-3 σis dysfunctional or that active CDC2 is uninhibited, perhaps due to methylated 14-3-3 σ or inhibition of CDC2 degradation [42] We observed that of the 57 ovarian cancer cell lines represented in the RPPA, 28 cell lines (49%) showed high levels of 14-3-3 σ (Figure 6A) of which 16 cell lines (28%) also had increased P-RB, corresponding to the O_2_ insensitivity pattern we have described. Amongst these 16 cell lines, 6 also have increased levels of CDC2 while the remainder had decreased levels of CDC2. This suggests that this protein profile is not exclusive to the cell lines we originally identified and might be representative of a relatively common phenomenon. We therefore determined whether this O_2_-insensitive associated 14-3-3 σ/CDC2/P-RB protein profile is also observed in ovarian tumor samples. Using the same criteria as with the cell line RPPA, we examined 205 ovarian tumor specimens using RPPA. This analysis revealed that 27% of ovarian tumors (56) had elevated levels of both 14-3-3 σ and P-RB, and amongst these, 34 also had elevated levels of CDC2 expression (Figure 6B). These results are very comparable with the RPPA analysis of the ovarian cancer cell lines (Figure 6A).

Ovarian cancer has a poor survival rate and this is often associated with metastatic progression [43]. The O_2_-insensitive associated 14-3-3 σ/CDC2/P-RB protein profile suggests an unrestricted G2/M control in response to changes in O_2_ levels, such as a migrating or metastatic cancer would encounter. Therefore, it is possible that this protein profile is associated with poor prognosis. Using the O_2_-insensitive associated protein profile (high P-RB with either high 14-3-3 σ or high CDC2) we identified 47 of 158 tumors with associated clinical data. A Kaplan-Meier survival estimate shows that patients with the O_2_-insensitive associated protein profile have a poor survival outcome (less than 90 months compared to 200 months observed otherwise, *p* = 0.016, Figure 6C). Altogether it appears that the O_2_-insensitive associated protein profile suggests that unrestricted G2/M accompanies a substantial proportion of ovarian cancer cells and primary tumor samples. Further, this O_2_-insensitive profile is associated with poor prognosis for this disease.

### Elevated 14-3-3 σ expression in metastatic ovarian tumors

**Figure 7:**
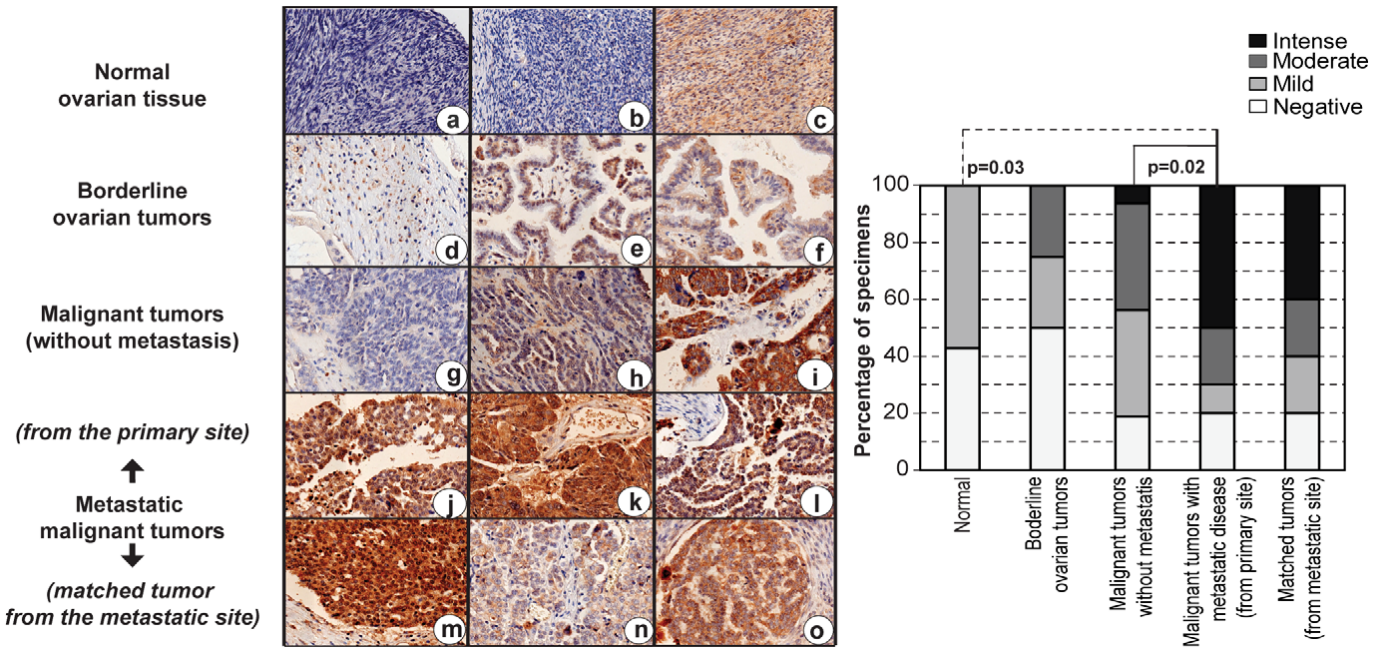
14-3-3 σ expression and ovarian tumor metastasis. Immunohistochemical analysis of 14-3-3 σ in ovarian tissues show negative (hematoxylin stained blue nucleus) to diffuse staining pattern for 14-3-3 σ (brown) in normal ovarian tissues (a–c), and a moderate increase in the staining intensity localized to the cytoplasm is observed in the borderline ovarian tumors (d–f). In the malignant tumors without any metastatic disease at diagnosis, 14-3-3 σ expression was either absent (g), or stained at moderate to intense levels (h–i), with occasional nuclear staining (i). Intense nuclear and cytoplasmic staining for 14-3-3 σ was observed in ovarian tumors with metastatic disease, obtained from the primary site of the disease, and a moderate to intense staining for 14-3-3 σ in the cytoplasm or both nucleus and cytoplasm of the corresponding tumors obtained from the metastatic site was observed [site of metastasis - (m) appendix, (n) lymph node and (o) omentum]. The quantitative relationship between 14-3-3 σ expression and various stages of ovarian cancer progression is represented in the bar graph, and the statistical analysis for correlation of expression with pathological grades were determined by a Fisher’s exact test.

Having observed that the O_2_-insensitive associated protein profile (high P-RB with either high 14-3-3 σ or high CDC2) is both relatively common in ovarian cancer and associated with poor prognosis, we went on to determine directly whether metastatic ovarian tumors exhibit an overt 14-3-3 σ signature. Of note, the ovarian tumors represented in the ovarian tumor RPPA are from primary sites and thus do not necessarily provide an accurate representation of the protein profile in the metastatic cancer. We therefore expect that metastatic tumors or primary tumors that give rise to metastatic tumors will exhibit a more overt 14-3-3 σ signature than primary tumors. In fact, an increased expression of 14-3-3 σ has been previously reported with other tumors [44] and a functional involvement for 14-3-3 σ in metastatic disease is known [45], [46]. We analyzed 14-3-3 σ expression using immunohistochemistry on paraffin embedded tissues obtained from 10 different metastatic ovarian tumors and their corresponding primary site tumors. We consistently observed intense immunostaining of 14-3-3 σ in 8/10 metastatic tumors and the corresponding primary tumors (Figure 7*j*–*l*). In contrast, the primary tumors without metastasis at diagnosis showed moderate immunostaining for 14-3-3 σ, and occasionally intense staining was also noted (Figure 7*i*). Borderline tumors showed a mild to moderate staining pattern for 14-3-3 σ, while in normal tissues, protein levels were absent or diffusely present (Figure 7 *a–c*). Increased expression of 14-3-3 σ in the metastatic primary tumors compared to normal tissue or malignant tumors without metastasis were observed to be statistically significant by the Fisher’s exact test (Figure 7, Bar Graph). The high level of 14-3-3 σ expression offers the first indication of the manner in which regulation of G2/M may be dysfunctional in these tumors.

Over-expression of 14-3-3 σ in metastatic disease is not unexpected and has been previously noted [45], [46], [47]. However, we speculate the reason for this association is due to a loss of O_2_-sensitivity and that this provides a selective advantage for metastatic progression. Our conclusion is that O_2_-sensitive and insensitive patterns of 14-3-3 σ and CDC2 expression are readily detectable and common to cancer cells, regardless of whether they are grown *in vivo* or *in vitro*. Further, these expression patterns may have prognostic implications, but additional experiments will be required to confirm the mechanistic relevance of O_2_-sensitivity in the clinical progression of cancer.

## Discussion

There is an increasing interest to study cell biology under the context of physiological O_2_ levels. Investigations with primary mouse embryonic fibroblasts comparing the effects of physiological (3%) and ambient (21%) oxygen, show that 21% O_2_ causes increased oxidative stress and induces senescence [4]. Several studies conducted with embryonic stem (ES) cells reported that characteristic stem cell properties are preserved only when ES cells are maintained under physiological O_2_- ES cells otherwise differentiate under ambient O_2_ as reviewed in [2]. This prompted us to investigate the effects of physiological (3%) and ambient (21%) oxygen in the context of cancer. With A2780 ovarian cancer cells grown under 21% or 3% O_2_, a 20% growth suppression was observed with 21% O_2_ by three days (Figure 2) and although the proportional changes to cell cycle profile appear small, they were significant (Table 1). The accumulated effect of these differences in proliferation and cell cycle resulted in a 2.6 fold difference to the growth of the cancer cells by 12 days in the presence of different O_2_ concentrations (Figure 1). This observation demonstrates that standard tissue culture conditions may adversely impact the *in vitro* proliferation of cancer, which is primarily a disease of proliferation. Previous studies compared the growth of primary mouse embryonic fibroblast cells [4], adult human fibroblasts [48] and human cancer cells [8] grown under physiological (3–5%) or ambient (21%) O_2_ and observed increased cell proliferation under physiological O_2_. In this study, we observed similar effects with ovarian cancer cells (A2780, OVCAR5, OVCAR8 and HOC8 - Figure 2), however other cells lines failed to respond to O_2_ concentration (HeyA8 and SKOV3) (Figure 2). These proliferative responses to O_2_ seem to affect all phases of the cell cycle, particularly the G1 and S phases of cell cycle, in all cell lines. However, only the G2 phase was affected in cell lines which displayed proliferative response to 3% O_2_ (Table 1), suggesting the possibility that the G2 phase transition of the cell cycle is crucial for regulating proliferation in response to differences in 3% O_2_ levels. A change in the G2 phase in response to O_2_ levels was reported in only one other study performed with Fanconi anemia (FA) cell lines [49]. Analogous to our study, the experiments with FA cells demonstrated a characteristic G2 delay with standard tissue culture conditions (20% O_2_), but a reduced proportion of cells in G2 and increased proliferation when cultured at 5% O_2_ [49]. Furthermore, growth of different human fibroblast cells under physiological O_2_ has also been observed to be accompanied by a reduction in the G2 cell population [27], [48]. Overall, it appears that the G2 phase is the most O_2_-sensitive phase of the cell cycle. Exploring the possible molecular mechanisms that render ovarian cancer cells either sensitive or insensitive to oxygen has clearly demonstrated that it is 14-3-3 σ and its inability to control CDC2 dependent G2/M transition in response to O_2_ levels that results in oxygen-insensitive cell lines. Although expression of 14-3-3 σ is regulated by p53 [25], we observed no difference in the levels of p53 expression under different oxygen concentrations (Figure S1), suggesting that the involvement of 14-3-3 σ in O_2_-sensitivity is independent of p53. If the decrease in 14-3-3 σ is associated with oxygen-sensitive increase in proliferation, then silencing the expression of 14-3-3 σ in oxygen-insensitive cell lines should restore proliferative sensitivity to oxygen. In fact, our experiments show that RNAi mediated silencing of 14-3-3 σ in HeyA8 cells restored oxygen sensitivity (Figure 5E) and in a converse experiment, over-expression of 14-3-3 σ abolished oxygen sensitivity in the A2780 cell line (Figure 5D). This suggests that high levels of 14-3-3 σ protein is sufficient to restrict the regulation of CDC2 mediated G2/M progression. The cytoplasmic restriction of overexpressed 14-3-3 σ in the O_2_-insenstive HeyA8 cells provides the first indication for the possible mechanistic basis of this dysregulation (Figure 5A). Other reports also show preferential changes to cellular localization of 14-3-3 σ during different phases of the cell cycle [50], suggesting that cell cycle changes observed with oxygen could be relevant to the 14-3-3 σ localization and pattern in our experiments. Furthermore, 14-3-3 σ is actively exported out of nucleus by CRM1, [51], a nuclear protein that is frequently over-expressed in ovarian cancer [52]. A host of other factors such as, BRCA1, p63 and estrogen induced zinc finger protein (EFP) are also known to regulate the levels of 14-3-3 σ [53]. Therefore, it is possible that 14-3-3 σ expression and its cellular distribution could be influenced by several factors, independent of p53 (as must be the situation in the O_2_ insensitive p53 null cell line SKOV3).

The differences in O_2_-sensitivity and, consequently, cell proliferation is most important when trying to recapitulate *in vivo* responses where physiological O_2_ tensions vary from 2.7–5% in the interstitial space (where many cancer cells reside) to 14.7% in the arterial circulation and lung [18]. Thus, it is reasonable to predict that if O_2_-sensitive cancer cells were to dislodge from a primary interstitial space and migrate to the lungs via blood circulation, the increased O_2_ concentration would restrict proliferation. In contrast, we speculate that oxygen insensitive cancer cells would have a selective advantage compared to sensitive ones, being better able to thrive in the conditions of increased oxygen concentration. In fact, 14-3-3 σ is frequently over-expressed in many thyroid [54], colorectal [55] and prostate [56] tumors, and is also a potential target for therapeutic modulation [55], [56]. Our results provide one rationale for selecting the cancers best suited for 14-3-3 σ targeted therapy. Oxygen insensitivity observed in HeyA8 or SKOV3 is less likely an adaptation to *in vitro* growth conditions because transient over-expression of 14-3-3 σ renders O_2_-sensitive A2780 cell line insensitive to increased levels of O_2_ (Figure 5D), and over-expression of 14-3-3 σ is observable in primary tumors with metastatic potential (Figure 7). Oxygen sensitivity could therefore be an important factor in the context of metastatic spread of cancer because over-expression of 14-3-3 σ is frequently observed in metastatic cancers, including this study (Figure 7) and others (gastric [57], endometrial [58] and pancreatic [59]). However, epigenetic inactivation of 14-3-3 σ by gene methylation has also been reported to correlate with decreased expression of 14-3-3 σ in cancer progression [60] and metastasis of certain types of tumors [61]. Further, a correlation with a functional role for 14-3-3 σ in promoting tumor invasion and metastasis has also been demonstrated [45], [47], [62]. Taken together, there is ample evidence to support that over-expression of 14-3-3 σ is relevant to tumor metastasis and therefore, it is likely that O_2_ insensitivity associated with over-expression of 14-3-3 σ may have a pivotal role in metastatic dissemination of tumors. Further support to demonstrate the explicit role of 14-3-3 σ in *in vivo* O_2_ sensitivity and its relevance to metastasis would require experiments with animal models.

In conclusion, there are many advantages to studying cancer biology under physiological O_2_. In fact, compared to cell propagation under physiological O_2_, ambient O_2_ levels are expected to result in oxidative stress [4], mutation proneness and persistence of transformation [63]. In this context, we have demonstrated that growing cancer cells *in vitro* at low physiological O_2_ (not hypoxia), compared with ambient (21%) O_2_ is a prudent approach to identify and understand some of the behavioral diversity observed in cancer.

## Materials and Methods

### Cell culture and Transfection

Ovarian cancer cells were grown in RPMI 1640 (A2780, OVCAR5, OVCAR8, SKOV3) or DMEM (HeyA8 and HOC8) supplemented with 10% heat inactivated Fetal Bovine Serum (Sigma Aldrich, St.Louis, MO, Cat# F6178) and 200 units of penicillin/streptomycin and 0.5 µg amphotericin-B. Transfection was performed using Amaxa Nucleofector technology (Lonza) as described previously [64]. Plasmid pcDNA 3.0 HA 14-3-3 σ was obtained from Addgene (plasmid 11946 [65]) and pcDNA 3.0 HA empty vector was a gift from Dr. Y. Shiio, UTHSCSA. 14-3-3 σ siRNA and non-targeting dsRNA were purchased from Dharmacon. For oxygen exposures we used Forma Series II 3110 water-jacketed multigas incubator (Thermo Fisher scientific, Waltham, MA) with built-in CO_2_ and O_2_ monitors and controllers. To maintain 3% O_2_, the incubator received an additional supply of nitrogen gas.

### Cell proliferation

Cell proliferation was determined using Celltitre-Glo (Promega, Madison, WI) per manufacturer instructions, as described previously [64]. Cells were seeded to a final density of 100, 200 or 400 cells per well in a 384 well plate containing 40 µl of growth medium consisting of 2%, 6% or 10% FBS and antibiotics. Plates were then placed in a humid chamber and returned to the incubators of appropriate oxygen pressure. After 3 days of incubation, the number of cells present per well was measured using Celltitre-Glo reagent, as described previously [64]. The number of cells per well was determined using a standard curve based on ATP concentration, as recommended by the manufacturer.

### Mitotic Index

The number of mitotic cells were quantified based the method as described [66]. Briefly, 96 well collagen coated plates were used to seed cells at a final concentration of 1000 cells/well in their respective media. Cells were then incubated for three days at 37°C in 3% or 21% O_2_. Finally, cells were washed, resuspended in phosphate buffered saline and stained with DAPI, as described [66]. Images of stained cells were acquired using a Zeiss Axiovert 200M inverted fluorescent microscope using 10X magnification and Openlab (PerkinElmer) image acquisition software. Using Image J, a set threshold for staining intensity was used to count the brightly stained nuclei, with obvious chromatin condensation and the mitotic index was determined based on the ratio of number of mitotic cells present in 1000 cells, as described [66].

### Protein isolation and Western blot analysis

Protein lysates and western blot analysis were preformed as previously described [64]. The immunoblots were probed with the appropriate dilutions of primary antibody and visualized using either Lumiglo (Cell signaling technology) or the ECL plus system (GE Healthcare) with the appropriate horseradish peroxidase-conjugated secondary antibody. The primary antibodies used were Phospho - p53 (Ser 15), total p53, Phospho - CDC2 (Tyr 15) and Total CDC2, Phospho - Cyclin B1 (Ser 133), total Cyclin B1, Phospho - CDC25C (Thr 160), total CDC25C, Phsopho RB (Ser 807/811), total RB, Phospho - WEE1 (Ser 642) and total WEE1 (Cell signaling technology), p21, 14-3-3 σ (Millipore) and β-actin (Abcam). Primary antibody dilutions were used as per manufacturer instructions. RB and WEE1 immunoblots were performed using 4–15% gradient gel (Criterion precast gel, Biorad).

### Flow Cytometry

Cells were trypsinized and seeded to a final density of 1×10^6^ cells per well in a 10 cm dish containing growth medium, antibiotics and appropriate concentrations of FBS. Dishes were then returned to the incubators set for the different oxygen conditions. Following three days of incubation, cells were harvested and prepared for FACS analysis as described previously [67]. Experiments were performed in triplicate. Stained cells were analyzed using a FACS Canto I (BD Biosciences) flow cytometer using an argon laser at 488 nm wavelength. Cell cycle analysis was performed using Modfit LT (version 3.2) software (Verity Software House).

### Quantification of M phase cells

The number of cells in M phase were quantified based on mitosis-specific histone H3 phosphorylation in the ovarian cancer cell lines using the Cellomics® Cell Cycle Kit I (Thermo Scientific) as per the manufacturer’s recommended protocol. Briefly, 96 well collagen coated plates were used to seed cells at a final concentration of 1000 cells/well in their respective media. Cells were then incubated for three days at 37°C in 3% or 21% O_2_. Control wells were treated with 1.5 µg/ml nocodazole (Sigma Aldrich) for 16 hours, fixed with 16% formaldehyde, permeabilized, blocked and stained with reagents consisting anti-phospho-histone H3 primary antibody, as per instructions provided in the kit. Stained cells were analyzed with a Zeiss Axiovert 200M inverted fluorescent microscope using 10X magnification and Openlab (PerkinElmer) image acquisition software. 100–250 cells per replicate were counted for phospho-histone H3 positive cells.

### Immunolocalization of 14-3-3 σ and CDC2

A2780 cells transfected with a 14-3-3 σ cDNA expression construct or HeyA8 cells transfected with 14-3-3 σ siRNA were seeded at a final density of 10^5^ cells per fibronectin (Sigma) coated 12.5 mm^2^ glass coverslip mounted in each well of a 12-well plate. Cells were maintained in complete growth medium supplemented with 10% fetal bovine serum and allowed to grow for three days in the presence of 21% or 3% oxygen. For the detection of 14-3-3 σ or CDC2 by immunofluorescence, cells were processed as described previously [68]. The primary antibodies used were mouse monoclonal 14-3-3 σ at 1.0 µg/mL (Upstate) and rabbit polyclonal total CDC2 at 1∶1000 (Cell Signaling). Following a PBS wash, the cells were incubated with secondary antibodies, goat anti-mouse AlexaFluor 488 and goat anti-rabbit AlexaFluor 568 (Invitrogen) at 1∶1000 dilution in blocking buffer for 1 hour at room temperature. Cells were then counterstained with DAPI (1∶3000 dilution in PBS) and mounted onto microscope slides using Fluoromount-G. Images were taken at 63X magnification using the Zeiss Axiovert 200M inverted fluorescent microscope and Openlab software (PerkinElmer).

### Reverse Phase Protein Array

Protein lysates from 57 cancer cell lines or 205 primary ovarian cancer tumors were spotted in RPPA slides and processed for expression analysis, as described previously [69], [70]. Data acquisition and processing were performed as described previously [69]. Ovarian cancer specimens were obtained from Gynecology Tumor Tissue Bank at MD Anderson Cancer Center, following approval from the Institutional Review Board (BT).

### Normalization and Clustering

log-transformed RPPA data was first examined to remove non ovarian cancer cell lines. We then examined all replicated representations from the same source as annotated to reduce down to 57 ovarian cancer cell lines or 205 patient samples (from each source) by taking the median protein expression level of all replicates. An additional cell-line specific normalization step was performed in which median expression levels for each protein was first determined and then subtracted from individual RPPA experiments. The anchored heatmap (termed after anchored over/under-expression orientation) was generated by requiring RB, 14-3-3 σ and CDC2 to be arranged from over-expressed to under-expressed recursively from the given cell-line order, but exact positions of each protein was determined by hierarchical clustering algorithm with Euclidean distance as similarity measure and average lineage from all cell-lines, as shown in Figure 6 A&B). Raw data obtained from RPPA for the expression of Phospho RB, 14-3-3 σ and CDC2 is provided in the supplementary tables (for ovarian cancer cell lines, see Table S2, and for ovarian cancer patient specimens, see Table S3)

### Immunohistochemistry

Tissue arrays (OV951-1) consisting of normal and malignant tissues from primary or metastatic sites were purchased from US Biomax Inc. Slides were processed for immunohistochemistry and analyzed, as described previously [71]. 14-3-3 σ (Upstate) was used at 1∶50 dilution for incubation with primary antibody and subsequent steps were performed using the Dako universal LSAB kit with DAB as described by the manufacturer.

### Statistical Analyses

To determine significant differences to proliferation under 3% or 21% O_2_, a Student *t-test* was performed, and *ANOVA* was performed to compare the different cell cycle profiles with the panel of ovarian cancer cell lines. Kaplan-Meier survival analysis with p-value determined with log-rank test was performed using MATLAB (Mathworks, Natick, MA) for RPPA data consisting patient specimens. For Kaplan-Meier survival analysis the data was censored based on patient's vital status. Statistical analysis for the correlation of 14-3-3 σ expression with the various pathological grades of ovarian tumors determined based on immunohistochemistry was analyzed by a Fisher’s exact test using R.

## Supporting Information

Figure S1 **Western blot analysis of phospho and total p53, and p21, which are major upstream regulators of G2/M cell cycle progression and the relevance to 21% or 3% O_2_ in ovarian cancer cells.** O_2_ insensitive cell lines are indicated by asterisk and italics.
(EPS)

Table S1 ***In vitro*** **cell doubling time for the ovarian cancer cell lines.**
(XLS)

Table S2 **Raw data from cell line RPPA for Phospho-Rb, p53, CDC2 and 14-3-3 σ expression.** The table contains following columns: 1) Unique ID, 2) Original cell-line or with treatment, 3) Cell-line's contributing Lab/source; 4) Cell type, 5) Cell-line name, 6–9) log2 transformed protein expression ratios (14-3-3 σ, CDC2, p-Rb, and p53) as the raw measurement provided by the MD Anders Cancer Center RPPA core facility.
(XLS)

Table S3 **Raw data from OVSS2 RPPA for Phospho Rb, CDC2 and 14-3-3 σ expression.** The table contains following columns: 1) Unique ID, 2) Tumor ID in various databases (DBs), 3) Tumor source institution; 4) patient age at diagnosis (in months), 5) tumor stage, 6) grade (HG: high grade, LG: low grade, empty: unknown), 7) Overall survival (in months), 8) Vital Status (0: alive, 1: dead), 9–12) log2 transformed protein expression ratios (14-3-3 σ, CDC2, p53 and p-Rb) as the raw measurement provided by the MD Anderson Cancer Center RPPA core facility.
(XLS)
